# Medicine in Dublin

**Published:** 1841-04

**Authors:** M. L. Linton


### THE WESTERN JOURNAL.

Vol. III.—No. IV.

LOUISVILLE, APRIL 1, 1841.

### MEDICINE IN DUBLIN. BY M. L. LINTON, M. D.

Dear Sir: I propose in the present letter some account of Medicine and Medical men in the Irish capital, which abounds more than any other city in Europe, of its size, in Hospitals, learned professors, and the other various requisites for medical instruction and improvement.

There are about twenty Hospitals in Dublin, of which the following are the principal: the Meath Hospital, the Richmond, Hardwick, and Whitworth Hospitals, Steven's Hospital, the Lying-in, Dean Swift's, Sir Patrick Dun's, a foundling establishment and a refuge for the incurable. I attended the Meath Hospital frequently. Drs. Graves and Stokes are principal physicians—they attend alternately. It was during the term of Dr. Stokes that my visits were made. Dr. Stokes is too well known both in Europe and America, to render it necessary that I should say any thing of him as a pathologist and therapeutician. He treated me with the utmost kindness—showed me all his cases—gave me his views of their pathology, and a plain account of his mode of treating them, with such other general views as he supposed would be interesting. Such attention as this, however, may be expected of the profession in general in Dublin, by any American physician, or perhaps I should say, by any visiting physician.

The prevailing disease in his wards was Typhus Fever. This, Dr.

Stokes considers, in opposition to Louis and the French pathologists generally, an essential or idiopathic disease not referable to any local lesion. He regards it as a disease which naturally tends to spontaneous resolution or cure, but which in its progress greatly exhausts the vital powers. Hence, his treatment is of a sustaining character, regardless, in a great degree, of the local irritations by which the disease is often complicated. Concomitantly with the wine, (for this is the stimulant principally used) he employs blistering, cupping and other revulsives, and evacuants, according to the local indications. He has lately instituted a series of experiments and observations on the effects of wine in typhus. He generally commenced its administration early, and continued it liberally, often giving as much as twenty-four ounces per diem.; the result has been eminently satisfactory, nearly all the patients having recovered. It is a general rule to which the Doctor subscribes, that when the pulse becomes slower and fuller, after the use of the wine, its utility and further administration are indicated. This rule applies as well, however, to other febrile diseases as to typhus. The Doctor has sought for another rule founded on the state of the heart. It is a fact, says he, that in some cases the heart acts violently when the pulse is very weak, and the converse, viz., that the heart is frequently found to act very feebly without a corresponding weakness of pulse. Now, it is in this latter condition, when the first sound of the heart is almost inaudible, and its corresponding impulse (the contraction of the ventricles) almost or quite imperceptible, that the use of wine, in liberal quantities, has been followed by the most remarkable success, restoring the sounds and impulses of the heart, and bringing the pulse into harmony with them—a condition quickly followed by convalescence. In opposite cases, in which the action of the heart is violent, the wine is not indicated, and if given, will aggravate the symptoms. The rule is to wait until the heart subsides to the point alluded to, and then commence the wine. Indeed, in most cases, the heart acts violently *at first*, but it *most generally* succumbs in a few days, and it should be closely watched in order that the wine may be brought to its aid, as soon as it gives signs of exhaustion. Wine was not the only sustaining agent relied on in these experiments—soups of a nutritious quality, were also resorted to, and in the latter stages of extremely bad cases, opium, musk, and ether were called in as adjuvants. This rule is perfectly practicable even by the tyro in Medicine, any one can estimate the sounds and impulses of the heart, and if it is as infallable as Dr. S. thinks it is, it is an invaluable addition to the science and art of healing. Dr. S. has observed in many cases the follicular ulcerations or dothinenteritis of the French, but he does not regard this condition of the intestines necessary to the constitution of the disease, as do most continental writers. I have already remarked that he regards it as an essential malady, and one that greatly exhausts the powers of the system. His object in the administration of wine is to sustain nature until she can rid the system of the disease, or, in other words, to use his own language, "*to cure the patient by keeping him from dying!*" Or, to select another set of words, to express the same idea, "to prevent the system from succumbing until the disease passes through its stages and subsides in virtue of the laws by which it is governed. It is a remark of Dr. Graves, that he wishes inscribed on his tomb the fact that he '*feeds fever*.' These doctrines differ scarcely at all from those which the Father of Medicine taught more than two thousand years ago, and yet Drs. Graves and Stokes are not one step behind the march of modern medicine. I state on the authority of the latter, that the debilitated state of the heart already alluded to in Typhus, is connected with a softened condition of that organ. Amongst the interesting cases in the medical wards, I saw a case of aortal aneurism; in this case Dr. S. informed me that he found generous feeding much better than rigid dieting.

The means which he uses in the treatment of paralysis are strychnine and electro-puncturation, with the kind of regimen which the circumstances of each individual case demands. In neuralgia the preparations of iron and the narcotic vegetable extracts seem to be his favorite remedies.

A host of eminent surgeons attend the Richmond Hospital; amongst whom I may mention Carmichael, O'Bierne, Adams, and M'Donnell; most of them are well known as authors. I saw Mr. Adams operate several times for club-foot, and once for wry neck. In the former, only the tendo achilles was divided, though they were cases in which the Parisians would have, in addition, divided other tendons. Mr. A. remarked that in fractures where the reunion of the bones was tardy, slight mercurialization would often bring it about. I should suppose that such might be the fact in cases in which there was a pre-existent syphilitic taint. He also showed me a case which he called '*mental syphilis*.' The patient labored under the melancholy belief, that the syphilitic virus was incorporated with his very being, when in truth he was entirely cured. This is not an uncommon sequent of that wretched disease; and every practitioner, has observed it, but they are indebted to Mr. Adams for its name. Though I reluctantly yield the palm, for Dr. Polin and myself called a similar case "*clap of the conscience*," many years ago!!

Lepra vulgaris has been treated successfully in this Hospital by small but gradually increased doses of the tinctura lyttœ, and repeated small bleedings. For Cirsocele the ablation of a part of the scrotum is a common remedy. This operation, which is quite safe and simple, converts the scrotum into a first-rate suspensory bandage. I have understood that Dr. Richardson, of Louisville, has resorted to it with success in at least one of his cases.

The Whitworth Hospital is devoted to chronic diseases. Indeed nearly all the cases were pulmonary consumption. I had not thought that phthisis was so prevalent in Ireland. But when the coldness and dampness of the climate is considered, as well as the destitution of all the comforts of life under which the poorer class labor, the fact of its prevalence is not at all surprising. Typhus fever and conpsumption are the scourges of the houseless, half clad, and half starved poor of oppressed Ireland.

The Hardwick is a Fever Hospital—most of the cases were Typhus. Upon inquiry I found that the treatment was 'expectant,' little being used but the spts. mindereri, with counter irritation, &c. for local symptoms, and wine for the latter stage of the disease. This was the treatment of Sr Philip Crampton, who is celebrated for his success in Typhus.

Stephen's Hospital is perhaps the most extensive in Dublin.—Cusack and Colles are the principal surgeons; Sir Henry Marsh is one of the physicians. It was here that I first saw the operation for strabismus. The obliquity was extreme and the operation did not succeed well. I saw the patient on the day after, in company with Mr. Cusack, who was the operator, and he regarded it as a failure. It was Mr. Colles' opinion that, after the operation, the healthy eye should be kept covered, in order that the consequently increased necessity for action on the part of the diseased one, should assist in rectifying its axis. This notion was acted upon but without effect.

I saw in this Hospital a great many cases of diseased bones, especially hip joint diseases, but could learn nothing in addition to what is generally known of their treatment. What do you do in such cases, asked I of Mr. Cusack. "What can we do?" was his laconic and honest reply. It is generally known that Mr. O'Bierne has succeeded in curing this disease in its *early stages*, by mercurialization. Cusack and Colles stand at the head of the surgical corps in Dublin. I have some personal acquaintance with the former, and should I hereafter meet with anything from his pen in favor of any method of treating disease, I should attribute to it more than ordinary weight. He writes but little, "because," says he, "my experience furnishes evidence for and against most of the methods of treating all diseases. Most medical men," said he, "publish only their favorable cases." In walking the wards with him, I saw several cases of puerperal fever. Upon enquiry I found that they had been brought from the Lying-in Hospital, in Sackvelle street, on account of a puerperal epidemic that was raging there at the time, and depopulating the entire establisment. I remarked, that according to my reading, but few women died of that disease, in the Lying-in Hospital, under the able supervision of Drs. Collins and Kenneday. "Ah," replied he, "these are the unpublished cases." The Lying-in Hospital alluded to, is the finest and most fashionably situated asylum in the city. I visited its wards, but one-half of the few that inhabited them were dying with puerperal fever. The treatment in general, venesect., counter irritation, and mercurials. In the present epidemic nothing seemed of any avail.

There are not less than ten faculties in Dublin, nor are these the only organs of oral instruction. Lectures are delivered at all the Hospitals, on medicine and surgery; and when the number of faculties, Hospitals and eminently distinguished anatomists, pathologists, and therapeuticians are considered, the assertion that the Irish Metropolis is second only to the French as a medical school, will not be contested.

I shall here close my remarks on the Dublin school. I might go on with a notice of the remaining Hospitals, and of the various faculties, but it would be of no practical utility. I shall devote the space that remains of this letter, to a few remarks on the Edinburgh and Glasgow schools.

As a medical school, Edinburgh stands deservedly as high as it ever did, and why should it not? when were there more learned professors in its faculty than Sir C. Bell, Christison, Hamilton, Syme, and the present Munro? Other schools have surpassed it, but it has not declined in merit. It is as great as ever *absolutely*, but not *relatively*.

Sir C. Bell was absent, on a visit to the continent, when I was there, but I had the pleasure of following the clinical professor of surgery, Mr. Syme, through the wards of the Royal Infirmary. He had just performed the operation for strabismus, with success. He was then treating some cases of urethral stricture, by the frequent introduction of a metalic sound, his object being to irritate the strictured part, and thus cause its ulceration or rather ulcerative absorption. I suppose it was on the principle, that what is caused by irritation, may be cured by irritating. I saw, also, some cases of gangrene of the extremities. (Gangrena senilis.) These cases were treated by rigid dieting, and the antiphlogistic regimen in every respect. "This treatment comports," said Syme, "with the low degree of vitality in the parts, and if you stimulate them to a higher action, sloughing will ensue." On the same principle, I suppose, that an ordinary degree of heat would cause the loss of a frozen limb.

Amongst the lecturers that I heard in Edingburgh was Dr. Traill. He attributed the first use of the stomach pump in cases of poisoning to "Dr. Physick of America." The Royal Infirmary is the principal Hospital of Edinburgh. It is within fifty paces of the University buildings. The number of students, at present, in all the dapartments of the University is about 1,400.

The Glasgow school, also, possesses all the advantages which good Hospitals and learned faculties can bestow. Amongst the cases which I saw in the Royal Infirmary of that city, I will mention three. In two of them the skull had been extensively fractured and driven in, the operation of trephining was performed on both, and in both cerebral hernia supervened—in one case as large as a pigeon's egg, and in the other as large as the firt—in the former the patient recovered—in the latter there was paralysis of the side opposite that of the cerebral lesion, when I last heard from him, (three weeks after the operation.) In the third case, the foot and ankle had been crushed by a waggon wheel; upon consultation it was resolved to attempt saving the limb, but traumatic gangrene soon made its appearance—extended to the body, and the patient died, 'maugre,' deep scarifications, blisters and poultices.

*March*, 1841.

